# Influence of Pheromone-Baited Traps on Stink Bugs in Cotton

**DOI:** 10.1093/jisesa/iey140

**Published:** 2019-02-19

**Authors:** P Glynn Tillman, Ted E Cottrell

**Affiliations:** 1 Crop Protection and Management Research Laboratory, United States Department of Agriculture, Agricultural Research Service, Tifton, GA; 2 Southeastern Fruit and Tree Nut Research Laboratory, United States Department of Agriculture, Agricultural Research Service, Byron, GA

**Keywords:** *Euschistus*, *Chinavia*, *Nezara*, pheromone-baited trap, area of arrestment

## Abstract

Stink bugs (Hemiptera: Pentatomidae) are economic pests in fruit, vegetable, grain, and row crops worldwide. Pyramid traps baited with lures of stink bug aggregation pheromones capture these pests in the field, but stink bugs can congregate on plants near traps. Our specific objective was to examine the area of arrestment of stink bugs based on their density on cotton at different distances from pheromone-baited traps. We used lures of the aggregation pheromone of *Euschistus* spp., methyl (2*E,*4*Z*)-2,4-decadienoate (MDD), and *Plautia stali* Scott, methyl [2*E,*4*E,*6*Z*]-2,4,6-decatrienoate (MDT). Overall, *Euschistus servus* (Say), *Euschistus tristigmus* (Say), *Chinavia hilaris* (Say), and *Nezara viridula* (L.) were the main stink bug species on cotton. Over the 3-yr study, adult stink bug density was significantly higher on the row of cotton immediately adjacent to a pheromone-baited trap than on the second and third row from the trap. Stink bug density was significantly lower on the seventh cotton row beyond the trap in 2015, on the fourth, eighth, and 16th rows in 2017, and on the fourth and eighth rows in 2018 compared to the two or three rows nearest the trap. These results indicate that adult stink bugs congregated mainly on the three cotton rows (2.73 m in width) nearest a trap. Management strategies utilizing pheromone-baited traps for stink bug control, such as trap cropping in combination with traps, should take into consideration this area of arrestment.

Phytophagous stink bugs (Hemiptera: Pentatomidae) are serious pests responsible for millions of dollars in losses and cost of control in fruit, vegetable, grain, and row crops (McPherson and McPherson 2000). For example, 166,520 bales of cotton nationwide were estimated lost due to stink bug pests in 2016 (Williams 2017). *Euschistus servus* (Say), *Euschistus tristigmus* (Say), *Nezara viridula* (L.), and *Chinavia hilaris* (Say) are the primary stink bug pests of cotton across the coastal plain of the southeastern United States, but other stink bug species, including *Euschistus quadrator* Rolston, *E. ictericus* (L.), and *Thyanta custator accerra* McAtee, are also pests of cotton (Bundy and McPherson 2000, McPherson and McPherson 2000). Stink bugs feed on developing cotton seeds and lint which can cause shedding of young bolls, yellowing of lint, yield reduction, and transmission of the bacterial pathogen *Pantoea agglomerans*, which can damage seed and lint (Barbour et al. 1990, Medrano et al. 2009).

Several economically important stink bug species produce an aggregation pheromone where both sexes congregate near the source of the pheromone. Aldrich et al. (1991) identified the major component of the Nearctic *Euschistus* spp. male-specific aggregation pheromone, methyl (2*E*, 4*Z)-*2,4*-*decadienoate (MDD), which attracts males, females, and nymphs of *E. servus* and other *Euschistus* spp. in the field. The two major components of the male-produced pheromone of *N. viridula* were identified as *trans*-(*Z*)-(1*S*,2*R*,4*S*)-epoxybisabolene and the corresponding *cis*-(*Z*)-(1*R*,2*S*,4*S*)-epoxybisabolene in a 3:1 ratio (Aldrich et al. 1987, Baker et al. 1987). These two components also are produced by male *C. hilaris*, but in a 19:1 ratio (Aldrich et al. 1989, McBrien et al. 2001). Sugie et al. (1996) identified the male-produced aggregation pheromone of *Plautia stali* Scott, methyl (2*E,*4*E,6Z*)-2,4,6-decatrienoate (MDT), which is attractive to both sexes of this stink bug species. Unfortunately, lures with the reported pheromones for *N. viridula* and *C. hilaris* are not commercially available. However, *C. hilaris* is cross-attracted to MDT under field conditions (Aldrich et al. 2007, Tillman et al. 2010).

A pyramid trap designed by Tedders and Wood (1994) to monitor the pecan weevil, *Curculio caryae* G. H. Horn (Coleoptera: Curculionidae), was modified by Mizell and Tedders (1995) to capture stink bugs. Several studies have shown that pyramid traps baited with commercially available MDD lures effectively capture adults of *Euschistus* spp., including *E. servus*, *E. tristigmus*, *E. ictericus*, *E. quadrator*, *E. politus* Uhler, and *E. conspersus* (Uhler), in the field (Mizell and Tedders 1995, Cottrell et al. 2000, Johnson et al. 2002, Leskey and Hogmire 2005, Tillman and Cottrell 2016a). Pyramid traps baited with MDD also capture *C. hilaris* and *N. viridula* in peanut and cotton field edges (Tillman and Cottrell 2016a).

The male-produced aggregation pheromone concentrates both sexes of stink bug adults in a limited area. Thus, stink bugs are sometimes present on plants near pheromone-baited traps (Aldrich et al. 1991, James et al. 1996, Krupke et al. 2001). For example, *E. conspersus* and *E. servus* aggregated and mated on mullein plants either baited with pheromone lures or in plots containing lures (Krupke et al. 2001, Leskey and Hogmire 2007). On many occasions, we have observed stink bug adults congregating on cotton near MDD pheromone-baited traps. Thus, our specific objective for this study was to examine the area of arrestment of stink bugs based on their density on cotton at various distances from traps baited with MDD and MDT.

## Materials and Methods

### Study Sites

In 2015, the trap test was conducted in cotton (Deltapine 1252 variety) at four field sites in Irwin County, GA: Jacob (31°56′ 37.00″N 83°30′ 05.54″W), Grove (31°55′ 81.54″N 83°29′ 91.03″W), Pecan (31°60′ 42.17″N 83°27′ 13.49″W), and Clark (31°60′ 69.83″N 83°27′ 54.59″W). In 2017, the trap test was conducted in cotton (Deltapine 1646 variety) at two field sites: Jacob and Dogwood (31°54′ 90.46″N 83°29′ 34.47″W). Also, cotton without traps was sampled at two field sites: Redbarn (31°55′ 44.22″N 83°31′ 35.75″W; Deltapine 1646 cotton variety) and Gray (31°57′ 17.55″N 83°28′ 77.39″W; Deltapine 1555 cotton variety). In 2018, the trap test was conducted in cotton (Deltapine 1555 cotton variety) at the House field site (31°32′ 57.99″N 83°19′ 16.86″W). Each site was approximately 12 ha except for the Dogwood, Redbarn, and House sites which were approximately 22 ha. In 2015, at the Jacob and Grove sites, cotton was planted on 26 May and on 29 May at the Pecan and Clark sites. In 2017, the Dogwood site was planted on 26 May, the Jacob and Gray sites were planted on 27 May, and the Redbarn site was planted on 30 May. In 2018, the House site was planted on 7 June. Except for the Redbarn site, the 2015 and 2017 sites occurred mainly in areas where peanut and cotton fields were closely associated. In 2018 cotton was not grown near peanut, but elderberry (*Sambucus nigra* subsp. *canadensis* [L.] R. Bolli) grew in surrounding woodlands and a 4-ha corn field was present near one field edge. For each field, a 12-row (10.97 m) swath was planted around the edges of the field with interior rows perpendicular to these rows. Row spacing was 0.91 m.

### Cotton Sampling Procedures

Treatment threshold for cotton is set at 20% internal boll injury the second week of flower, 10–15% internal boll injury the third through fifth weeks of flower, 20% internal boll injury the sixth week of flower, and 30% internal boll injury the seventh week of flower (Collins 2015). Therefore, cotton was examined for the presence of stink bugs from the second through seventh week of flower. For each cotton sample, all plants within a 1.83-m length of row were shaken over a drop cloth, and the number of stink bugs was recorded. Boll injury was assessed by examining a boll (~2.5 cm in diameter) at each sampling site (see below) for internal injury (i.e., warts and stained lint) caused by stink bugs as described by Bundy et al. (2000). Voucher insects are stored in the USDA, ARS, Crop Protection & Management Research Laboratory in Tifton, GA.

### Stink Bug Traps and Pheromone Lures

A stink bug trap consisted of an insect-collecting device made from a 2.8-liter clear plastic PET jar (United States Plastic Corp., Lima, OH) with a screw-cap lid (10.2 mm in diameter) seated atop a 1.22-m-tall yellow pyramid base (Cottrell et al. 2000, photo in Tillman and Cottrell 2017). The insect-collecting device was baited with a lure of the aggregation pheromone of *Euschistus* spp. (MDD) and one of *P. stali* (MDT). MDD was purchased from Degussa AG Fine Chemicals (Marl, Germany), and lures were produced following the procedures in Cottrell and Horton (2011). MDT lures were purchased from Agbio, Inc. (Westminster, CO). An insecticidal ear tag (10% λ-cyhalothrin and 13% piperonyl butoxide) (Saber extra insecticide ear tags, Sagebrush Tags, De Smet, SD) also was placed in this device to decrease stink bug escape (Cottrell 2001). Lures with the reported pheromones for *N. viridula* and *C. hilaris* could not be used because they currently are not commercially available. In 2015, traps were established on 13 and 14 July at the Grove and Jacob sites, respectively, and on 15 July at the Pecan and Clark fields. In 2017, traps were placed in the Jacob and Dogwood fields on 18 July. In 2018, traps were established at the House field on 8 August. Thereafter, lures were changed and insects collected on a weekly basis from the first through seventh week of flower (mid-July to early September) at the Jacob and Grove sites and from the first through sixth week of flower (mid-July to late August) at the Pecan and Clark sites during 2015, from the first through seventh week of flower (late July to mid-September) in the Jacob and Dogwood sites in 2017, and from the third through seventh week of flower (mid-August to mid-September) at the House site in 2018.

### Experimental Design

For cotton fields with traps, a pheromone-baited pyramid trap was placed equidistant between two interior rows. In 2015, traps were established in cotton on a transect at 15.24, 30.48, 60.96, and 121.91 m from the field edge using three transects at the Jacob and Pecan sites, four transects at the Clark site, and five transects at the Grove site. Cotton was sampled on the row of cotton immediately adjacent to a pheromone-baited trap and on the second and seventh rows from the trap. In 2017, traps were established on a transect at 15.24, 45.72, and 106.67 m from the field edge using 12 transects at the Jacob site and five transects at the Dogwood site. Cotton was sampled on row 1 near the trap and on rows 2, 4, 8, and 16 beyond the trap. For fields without traps in 2017, cotton was sampled on a single row on a transect at 15.24, 45.72, and 106.67 m from the field edge using 16 transects at the Redbarn site and four transects at the Gray site. In 2018, traps were established on a transect at 15.24, 45.72, and 106.67 m from the field edge using 10 transects at the House site. Cotton was sampled on row 1 near the trap and on rows 2, 3, 4, and 8 beyond the trap.

In 2015 and 2018, cotton was sampled at 0.91 (edge row 1), 1.83 (edge row 2), 4.57 (edge row 5), and 8.23 (edge row 9) m from the field edge directly in front of a trap transect and again at another field edge at least 22.9 from the field edge near traps. In 2015, three samples were taken on each edge row for 8 field edges per treatment (presence or absence of traps), and in 2018, a single sample was taken on each edge row for 7 field edges associated with traps and 10 field edges unassociated with traps.

### Data Analyses

All data were analyzed using SAS statistical software (SAS Institute, Inc. 2010, Cary, NC). Chi-square analyses were used to compare overall frequency of stink bug species in cotton (PROC FREQ). Seasonal means for the number of adult stink bugs per trap were calculated (PROC MEANS).

For cotton with traps, stink bug count data were modeled using a Poisson distribution. The analyses were done using PROC GLIMMIX. The KENWARD-ROGER option and the LINK=LOGIT function were used in the model statement. Model fit was evaluated by use of the chi-square and df statistic provided by PROC GLIMMIX (Littell et al. 2006). Fixed effects were cotton row adjacent to the trap, week, distance from the field edge, field (except for the House site in 2018), and row by week. Random effects were replicate and residual error. For each year, the row by week interaction was insignificant for density data; the interaction was dropped from the model which was rerun. In 2015 at the Grove site and in 2018 at the House site, the second week of flower was excluded from analyses because stink bugs were absent that week. In 2015, stink bugs were not detected on cotton at the Pecan and Clark sites on the sixth and seventh week of flower and at the Jacob site on the seventh week of flower so these weeks were excluded from data analyses. Means were back-transformed using the ILINK option in the LSMEANS statement and compared using Tukey’s honestly significant difference (HSD).

To determine if stink bug density on rows 4, 8, and 16 rows beyond a pheromone-baited trap in fields with traps was comparable to stink bug density on cotton in fields without traps in 2017, these stink bug count data were modeled using a Poisson distribution. The analyses were done using PROC GLIMMIX. The KENWARD-ROGER option and the LINK=LOGIT function were used in the model statement. Fixed effects were treatment (presence or absence of traps), distance from the field edge, and treatment by distance from the field edge. Random effects were replicate and residual error. The treatment by distance interaction was insignificant for density data; the interaction was dropped from the model which was rerun. Means were back-transformed using the ILINK option in the LSMEANS statement and compared using Tukey’s HSD.

To determine if stink bug density on cotton in field edge rows directly in front of a trap transect was comparable to stink bug density on cotton in field edges without traps in 2015 (Jacob and Grove sites only) and 2018, these stink bug count data were modeled using a Poisson distribution. The analyses were done using PROC GLIMMIX. The KENWARD-ROGER option and the LINK=LOGIT function were used in the model statement. In 2015, subsamples per row and rows per field edge were pooled. In 2018, rows per field edge were pooled. Fixed effects were treatment (presence or absence of traps), week, and treatment by week. Random effects were replicate and residual error. For the House site, the second week of flower was excluded from analyses because stink bugs were absent that week. For both years, the treatment by week interaction was insignificant for density data; the interaction was dropped from the model which was rerun. In 2018, week effect was insignificant and was dropped from the model which then was rerun. Means were back-transformed using the ILINK option in the LSMEANS statement and compared using Tukey’s HSD.

Boll injury data were modeled using a binomial distribution. The analyses were done using PROC GLIMMIX. The KENWARD-ROGER option and the LINK=LOGIT function were used in the model statement. The response variable was the number of injured cotton bolls divided by the total number of bolls by row by transect (samples were pooled by row by transect). Fixed effects were row, week, field (except for the House site in 2018), and row by week. Random effects were replicate and residual error. For each year, the row by week interaction was insignificant; the interaction was dropped from the model which was rerun. Means were back-transformed using the ILINK option in the LSMEANS statement and compared using Tukey’s HSD.

## Results

The same seven species of plant-feeding stink bugs were detected on cotton every year of the study (Table 1). *Euschistus tristigmus* was the predominate species in 2015, and *E. servus* was the predominant species in 2017. As a group *Euschistus* spp. were the major species in 2015 (85.6%) and 2017 (75.6%). In 2018, though, *C. hilaris*, *E. servus*, and *N. viridula* together were the most prevalent stink bug species (83.9%), and *Euschistus* spp. represented 41.4% of all stink bug species.

**Table 1.** Frequency of occurrence of stink bug species in cotton in 2015, 2017, and 2018

Year	Stink bug species	Frequency of occurrence	χ^2^	df	*P*
2015	*E. tristigmus*	63.2	265.3	6	<0.0001
	*E. servus*	18.4			
	*C. hilaris*	10.4			
	*E. quadrator*	3.2			
	*T. c. custator*	2.4			
	*N. viridula*	1.6			
	*E. ictericus*	0.8			
2017	*E. servus*	51.2	172.1	6	<0.0001
	*E. tristigmus*	19.9			
	*N. viridula*	12.2			
	*C. hilaris*	11.5			
	*E. ictericus*	3.8			
	*E. quadrator*, *T. c. custator*	0.7			
2018	*C. hilaris*	33.4	74.2	6	<0.0001
	*E. servus*	27.3			
	*N. viridula*	23.2			
	*E. tristigmus*	9.1			
	*E. ictericus*	4.0			
	*T. c. custator*	2.0			
	*E. quadrator*	1.0			

In 2015, density of stink bug adults on cotton associated with pheromone-baited traps was significantly influenced by row (*F*_2, 756_ = 26.46, *P* < 0.0001) and week (*F*_5, 756_ = 5.42, *P* < 0.0001) but not field (*F*_3, 556.4_ = 0.18, *P* = 0.9119) or distance from the field edge (*F*_3, 765_ = 0.73, *P* = 0.5329). Density was significantly greater on cotton row 1 next to a pyramid trap than the density two rows from the trap and significantly greater on row 2 than the density seven rows beyond the trap (Table 2). However, no significant row effect on boll injury (*F*_2, 223_ = 1.74, *P* = 0.1787) was observed. Stink bug density peaked on cotton the third and fourth weeks of flowering, but boll injury was not significantly affected by week (*F*_5, 223_ = 0.97, *P* = 0.4381) (Table 3). In addition, economic threshold was not reached any week for any site. Density of stink bug adults was similar for each distance into the field (Table 4). Density was similar for the Jacob (0.09 ± 0.03), Pecan (0.08 ± 0.04), Clark (0.07 ± 0.03), and Grove (0.08 ± 0.04) sites. No significant field effect on boll injury (*F*_3, 223_ = 1.81, *P* = 0.1463) was observed for the Jacob (0.75% ± 0.46), Pecan (2.14% ± 1.36), Clark (2.2% ± 1.0), and Grove (0.67% ± 0.35) sites. Overall mean (±SE) capture of stink bug adults per trap was 4.55 ± 0.18. A total of 1,891 stink bug adults were captured in 64 traps; 125 stink bug adults were detected on cotton.

**Table 2.** Least squares mean (±SE) for number of stink bug adults per 1.83-m length of row in cotton and cotton boll injury by row adjacent to a stink bug pheromone-baited pyramid trap in 2015, 2017, and 2018

Year	Row	No. in cotton	% Boll injury
2015	1	0.22 ± 0.09a	1.31 ± 0.64a
	2	0.08 ± 0.04b	0.72 ± 0.42a
	7	0.03 ± 0.01c	1.89 ± 0.86a
2017	1	0.21 ± 0.03a	4.32 ± 0.94a
	2	0.06 ± 0.01b	2.43 ± 0.84a
	4	0.03 ± 0.01c	3.68 ± 0.11a
	8	0.03 ± 0.01c	2.18 ± 0.79a
	16	0.02 ± 0.01c	2.68 ± 0.89a
2018	1	0.35 ± 0.05a	9.25 ± 1.75a
	2	0.15 ± 0.03b	11.43 ± 2.64a
	3	0.09 ± 0.02b	11.43 ± 2.64a
	4	0.03 ± 0.01c	5.23 ± 1.78ab
	8	0.01 ± 0.01c	2.29 ± 1.13b

For each year, least squares means followed by the same letter in the same column are not significantly different (Tukey’s HSD, α = 0.05).

In 2017, density of stink bug adults on cotton near pheromone-baited traps was significantly influenced by row (*F*_4, 1607_ = 24.59, *P* < 0.0001) and week (*F*_5, 1607_ = 3.92, *P* = 0.0015) but not field (*F*_1, 1607_ = 0.02, *P* = 0.8938) or distance from the field edge (*F*_2, 1607_ = 0.38, *P* = 0.6868). Density of stink bug adults was significantly greater on the row nearest a pyramid trap than on row 2 beyond the trap (Table 2). In addition, stink bug density was significantly higher on the second row of cotton compared to rows 4, 8, and 16. No significant row effect on boll injury (*F*_4, 1_ = 1.42, *P* = 0.552) was observed. Stink bug density peaked the fifth and sixth weeks of flowering (Table 3). Even though boll injury was not significantly different among weeks (*F*_5, 1_ = 4.32, *P* = 0.3492), the highest level of boll injury occurred the fifth week of flower. Economic threshold was not reached any week for each cotton field. Density of stink bug adults was similar for each distance into the field (Table 4). Density was similar for the Jacob (0.05 ± 0.01) and Dogwood (0.05 ± 0.01) sites. No significant field effect on boll injury (*F*_1, 130_ = 0.01, *P* = 0.9956) was observed for the Jacob (2.96% ± 0.56) and Dogwood (2.96% ± 0.78) sites. Overall mean (±SE) capture of stink bug adults per trap was 3.59 ± 0.19. A total of 1035 stink bug adults were captured in 36 traps; 131 stink bug adults were detected on cotton.

**Table 3.** Least squares mean (±SE) for number of stink bug adults per 1.83-m length of row in cotton and cotton boll injury by week for fields with stink bug pheromone-baited pyramid traps in 2015, 2017, and 2018

Year	Week of flower	No. in cotton	% Boll injury
2015	2	0.04 ± 0.02d	0.26 ± 0.27a
	3	0.16 ± 0.07ab	1.29 ± 0.074a
	4	0.21 ± 0.08a	1.56 ± 0.84a
	5	0.12 ± 0.05c	1.29 ± 0.73a
	6	0.07 ± 0.03cd	1.56 ± 0.84a
	7	0.03 ± 0.02d	2.98 ± 1.89a
2017	2	0.01 ± 0.01b	0.59 ± 0.42a
	3	0.07 ± 0.01a	6.0 ± 1.43a
	4	0.05 ± 0.01a	5.09 ± 1.31a
	5	0.08 ± 0.02a	6.92 ± 1.55a
	6	0.08 ± 0.02a	4.19 ± 1.18a
	7	0.05 ± 0.01a	1.19 ± 0.6a
2018	3	0.06 ± 0.02a	4.32 ± 1.47b
	4	0.05 ± 0.02a	3.34 ± 1.29b
	5	0.07 ± 0.02a	9.77 ± 2.27a
	6	0.08 ± 0.02a	10.28 ± 2.33a
	7	0.10 ± 0.02a	12.83 ± 2.61a

For each year, least squares means followed by the same letter in the same column are not significantly different (Tukey’s HSD, α = 0.05).

In 2017, density of stink bug adults was not significantly different (*F*_1, 1207_ = 0.31, *P* = 0.5763) on cotton rows 4, 8, and 16 beyond traps (0.03 ± 0.01) compared to density on cotton in fields without traps (0.02 ± 0.01). Density was not significantly influenced by trap distance (*F*_2, 1207_ = 0.13, *P* = 0.8742) but was similar for the 15.24 (0.03 ± 0.01), 45.72 (0.03 ± 0.01), and 106.67 m (0.02 ± 0.01) distances. Economic threshold was not met any week for each cotton field.

In 2018, density of stink bug adults on cotton near pheromone-baited traps was significantly influenced by row (*F*_4, 739_ = 15.56, *P* < 0.0001) but not week (*F*_4, 739_ = 1.32, *P* = 0.2624) or distance from the field edge (*F*_2, 739_ = 2.71, *P* = 0.0674). Density of stink bug adults was significantly greater on the row nearest a pyramid trap than on row 2 beyond the trap (Table 2). In addition, stink bug density was significantly higher on the second and third rows of cotton compared to rows 4 and 8. Over each year of the study, 92.0% of the *E. servus*, 91.3% of the *E. tristigmus*, 85.5% of the *C. hilaris*, 72.7% of the *N. viridula*, and 83.3% of the three remaining stink bug species, *E. ictericus*, *E. quadrator*, and *T. c. custator*, congregated on cotton rows 1 through 3. A significant row effect on boll injury (*F*_4, 241_ = 2.99, *P* = 0.0197) was observed. Boll injury was higher on rows 1, 2, and 3 than on row 8 but not on row 4. Boll injury was significantly different among weeks (*F*_4, 241_ = 4.17, *P* = 0.0028); the highest level of boll injury occurred the fifth through seventh weeks of flower (Table 3). Economic threshold was not reached any week. Density of stink bug adults was similar for each trap distance (Table 4). Overall mean (±SE) capture of stink bug adults per trap was 5.69 ± 0.29. A total of 854 stink bug adults were captured in 33 traps; 99 stink bug adults were detected on cotton.

**Table 4.** Least squares mean (±SE) for number of stink bug adults per 1.83-m length of row in cotton by distance from the field edge for fields with stink bug pheromone-baited pyramid traps in 2015, 2017, and 2018

Year	Distance (m)	No. in cotton
2015	15.24	0.07 ± 0.03a
	30.48	0.09 ± 0.04a
	60.96	0.08 ± 0.03a
	121.91	0.10 ± 0.04a
2017	15.24	0.04 ± 0.01a
	45.72	0.05 ± 0.01a
	106.67	0.05 ± 0.01a
2018	15.24	0.09 ± 0.02a
	45.72	0.06 ± 0.01a
	106.67	0.06 ± 0.02a

For each year, least squares means followed by the same letter in the same column are not significantly different (Tukey’s HSD, α = 0.05).

For cotton in field edges, in 2015 density of stink bug adults was significantly influenced by week (*F*_5, 98_ = 3.69, *P* = 0.0042) but not pheromone-baited traps (*F*_1, 98_ = 1.66, *P* = 0.2). Again in 2018, stink bug adult density on cotton in field edges was not by affected by pheromone-baited traps (*F*_1, 49_ = 2.38, *P* = 0.1296). In general, in 2015 stink bug density (mean number per sample ± SE) increased with cotton maturity (second week of flower, 0.12 ± 0.09; third week of flower, 0.49 ± 0.18; fourth week of flower, 0.56 ± 0.19; fifth week of flower, 0.82 ± 0.22; sixth week of flower, 1.34 ± 0.24; seventh week of flower, 0.68 ± 0.2). In both years, density of stink bug adults (mean number per sample ± SE) was similar in cotton field edges directly in front of a trap transect (2015, 0.46 ± 0.1; 2018, 0.86 ± 0.2) and those unassociated with traps (2015, 0.63 ± 0.12; 2018, 0.5 ± 0.13).

## Discussion

In this study, *E. tristigmus* and *E. servus* were the most frequently captured stink bug species in MDD-MDT pheromone-baited traps in cotton during 2015 and 2017. An earlier study was conducted to examine the attractiveness of the pheromone of *E. servus, N. viridula*, and *C. hilaris* to each stink bug species in field plots in Georgia (Tillman et al. 2010). Only MDD, either alone or in combination with MDT, was attractive to *E. servus*. Thus, it was not surprising that *E. servus* and *E. tristigmus* were highly attracted to traps baited with MDD in the current study. These two stink bug species were also the predominant ones in MDD-baited traps in pecan orchards in Florida and Georgia, apple and peach orchards in the mid-Atlantic area, and cotton and peanut field edges in areas where peanut and cotton are grown in nearby fields in Georgia (Yonce and Mizell 1997, Cottrell et al. 2000, Leskey and Hogmire 2005, Tillman and Cottrell 2016a).

In contrast with 2015 and 2017, during 2018, *C. hilaris*, *E. servus*, and *N. viridula* were the main stink bug species captured in traps. Tillman et al. (2010) showed that traps baited with the reported pheromone of *C. hilaris* failed to attract more *C. hilaris* than unbaited control traps. Furthermore, this stink bug species was not attracted to MDD or the *N. viridula* pheromone. Instead, *C. hilaris* was highly attracted to MDT indicating that MDT is an effective pheromone for capturing this stink bug species in traps. Elderberry is a non-crop host plant of *C. hilaris* and *E. servus* (Tillman and Cottrell 2016b). In 2018, these two stink bug species developed on elderberry in woodlands surrounding the House cotton field; *E. tristigmus* adults were observed feeding on elderberry fruit (P.G.T., unpublished data). An earlier study documented that *C. hilaris* and *E. servus* dispersed from elderberry into cotton at this site (Tillman and Cottrell 2016b). This is the likely explanation for the higher frequency of *C. hilaris* in traps in 2018 compared to 2015 and 2017.

In previous field experiments, capture of *N. viridula* was significantly higher in traps baited with lures of its reported pheromone compared to MDD or MDT pheromone-baited traps and unbaited control traps (Tillman et al. 2010). Thus, in 2018, trap capture was probably lower for *N. viridula* using traps baited only with MDD and MDT than if traps had been baited with the *N. viridula* pheromone. However, the current study was not focused on abundance per se but on arrestment behavior of stink bugs on cotton plants near traps, and 72.7% of the *N. viridula* in cotton congregated on plants near the MDD-MDT traps. *Nezara viridula* is likely cross-attracted to MDD, and perhaps to MDT, in traps in crop fields as indicated by its capture in MDD-baited traps in an earlier study (Tillman and Cottrell 2016a) and in MDD-MDT traps in the current study. Corn is a host plant of *N. viridula* and *E. servus* (Tillman 2011), and in 2018, both species developed on corn near cotton (P.G.T., unpublished data). In fact, an earlier study revealed that both species dispersed from corn into peanut and likely dispersed from corn into cotton (Tillman 2011). This may explain the higher frequency of *N. viridula* in traps during 2018 than during 2015 and 2017.

Pyramid traps baited with aggregation pheromone can be used as monitoring tools to assess the relative abundance and distribution of stink bugs in crops (Tillman and Cottrell 2017). However, stink bug adults can aggregate on plants near pheromone-baited traps. For example, *E. tristigmus*, *E. servus*, and *E. politus* were caught in traps or within 1 m of traps baited with synthetic attractants (Aldrich et al. 1991). Also, *Euschistus conspersus* was found more often on mullein plants associated with pheromone-baited traps than were actually caught in traps (Krupke et al. 2001). Consistently for each year of our study, density of stink bug adults was approximately three times higher on cotton immediately adjacent to a MDD-MDT pheromone-baited pyramid trap than the density on cotton two rows from the trap, and stink bug density was higher on rows 2 and 3 compared to that on rows 4, 7, 8, and 16. In 2017, density of stink bug adults on cotton rows 4, 8, and 16 beyond a trap and on cotton not associated with a trap was similar thus indicating that stink bug density on these rows was not influenced by the trap. It is more likely that stink bug presence on cotton rows 4, 8, and 16 from the trap was due to their dispersal into the cotton field in response to the deteriorating suitability of non-crop hosts surrounding cotton (Tillman and Cottrell 2016b). An edge effect in dispersal of stink bug adults occurs in cotton (Tillman et al. 2014). In the current study, density of stink bug adults in edges of cotton fields, directly in front of trap transects, was similar to density in edges of cotton fields not associated with traps. This strongly suggests that these pheromone-baited traps did not attract stink bug adults into the field.

Because stink bug density significantly dropped on cotton four rows from the trap, the area of arrestment of stink bugs near the pheromone-baited trap was likely no more than 2.74 m (three rows of cotton). Even though percentage species composition changed over the 3 yr of the study, each stink bug species congregated on cotton near a pheromone-baited trap and the area of arrestment was similar each year. Interestingly, this area of arrestment is within the range previously reported for the same, as well as other, stink bug species. Adult *E. servus* aggregated on mullein plants over a zone of 3.14 m^2^ around a MDD-baited trap (Leskey and Hogmire 2007). *Halyomorpha halys* (Stål) and native stink bugs, including *Murgantia histrionica* (Hahn), *Euschistus* spp., *C. hilaris*, and *N. viridula*, were attracted by the aggregation pheromone of *H. halys* and the MDT pheromone synergist and arrested on unbaited traps within a 2.5-m radius of a baited trap (Morrison et al. 2016).

With one exception, cotton boll injury was similar for each cotton row sampled indicating that concentration of stink bugs on cotton near a baited pheromone trap does not negatively influence boll damage. The exception was the lower stink bug density on row 8 beyond the trap compared to that on rows 1 through 3 in 2018. Nonetheless, the economic threshold was not met for any row near traps. These results indicate that stink bugs are likely arrested on cotton near the trap for a relatively short period of time before moving to the insect-collecting device where they are killed.

In addition to being used for monitoring the relative abundance of stink bugs, pheromone-baited traps can also be utilized to manage stink bugs by capturing and killing stink bugs in trap crops. Combining soybean, i.e., an attractive stink bug food source, with pheromone-baited traps can be an effective management tactic to trap and kill stink bugs within the trap crop throughout the cotton growing season (Tillman et al. 2015). In this earlier study, the traps were placed 0.91 or 1.81 m from cotton that was adjacent to soybean. Trap capture of *E. servus* and *C. hilaris* in soybean was high, but stink bug density in cotton was numerically, but not statistically, reduced with the addition of baited traps in the trap crop. This may have been due to the fact that the traps were too close to cotton, as demonstrated in the current study, to avoid congregation of stink bugs on the cotton rows closest to the trap crop. Therefore, pheromone-baited traps should be placed ≥3 m from the host crop. Also, combining traps with fruiting soybean was a significantly more effective management tactic than using stand-alone traps. Sargent et al. (2014) tested the assertion that MDT-baited traps can be used in home gardens to reduce damage by *H. halys* to tomato fruit. However, tomato fruit grown in gardens with traps sustained significantly more injury than fruit grown in gardens without traps. Perhaps the higher injury was due to congregation of stink bugs feeding on tomato near the traps.

Pheromone-baited traps can also be utilized to manage stink bugs by attracting them to a specific location where they are then killed, known as the ‘attract-and-kill’ approach. To assess whether this approach held promise for managing *H. halys*, Morrison et al. (2016) first determined that this pest was arrested on unbaited traps within a 2.5-m radius of a baited trap, and retention time was greater in association with a host plant (apple) which may be why trap capture was higher when traps were associated with a fruiting host plant in the above trap cropping study. In an experimental apple orchard, mortality of *H. halys* was high in baited attract-and-kill trees season-long with little damage to adjacent unbaited trees suggesting this approach may be very effective in managing this pest.

In summary, adult stink bugs congregated mainly on the three cotton rows (2.73-m in width) nearest a pheromone-baited trap. Management strategies utilizing pheromone-baited traps for stink bug control, such as trap cropping in combination with traps, should take into consideration the area of arrestment of stink bugs on plants near traps.

## References

[CIT0001] Aldrich J. R., Oliver J. E., Lusby W. R., Kochansky J. P., and Lockwood J. A. 1987 Pheromone strains of the cosmopolitan pest, *Nezara viridula* (Heteroptera: Pentatomidae). J. Exp. Zool. 244: 171–176.

[CIT0002] Aldrich J. R., Lusby W. R., Marron B. E., Nicolaou K. C., Hoffmann M. P., and Wilson L. T. 1989 Pheromone blends of green stink bugs and possible parasitoid selection. Naturwissenschaften 76: 173–175.

[CIT0003] Aldrich J. R., Hoffmann M. P., Kochansky J. P., Lusby W. R., Eger J. E., and Payne J. A. 1991 Identification and attractiveness of a major component for Nearctic *Euschistus* spp. stink bugs (Heteroptera: Pentatomidae). Environ. Entomol. 20: 477–483.

[CIT0004] Aldrich J. R., Khrimian A., and Camp M. J. 2007 Methyl 2,4,6-decatrienoates attract Stink bugs (Hemiptera: Heteroptera: Pentatomidae) and tachinid parasitoids. J. Chem. Ecol. 33: 801–815. 17334917 10.1007/s10886-007-9270-9

[CIT0005] Baker R., Borges M., Cooke N. G., and Herbert R. H. 1987 Identification and synthesis of (*Z*)-(1’S,3’R,4’S)(-)-2-(3’,4’-epoxy-4’-methylcyclohexyl)-6-methylhepta-2,5-diene, the sex pheromone of the southern green stink bug, *Nezara viridula* (L.). J. Chem. Soc. Chem. Commun. 1987: 414–116.

[CIT0006] Barbour K. S., Bradley J. R. Jr., and Bachelor J. S. 1990 Reduction in yield and quality of cotton damaged by green stink bug (Hemiptera: Pentatomidae). J. Econ. Entomol. 83: 842–845.

[CIT0007] Bundy C. S., and McPherson R. M. 2000 Dynamics and seasonal abundance of stink bugs (Heteroptera: Pentatomidae) in a cotton-soybean ecosystem. J. Econ. Entomol. 93: 697–706. 10902318 10.1603/0022-0493-93.3.697

[CIT0008] Bundy C. S., McPherson R. M., and Herzog G. A. 2000 An examination of the external and internal signs of cotton boll damage by stink bugs (Heteroptera: Pentatomidae). J. Entomol. Sci. 35: 402–410.

[CIT0009] Collins G., ed. 2015 The 2015 Georgia Cotton Production Guide, CSS-15-01. University of Georgia College of Agricultural and Environmental Sciences Cooperative Extension, Athens, GA.

[CIT0010] Cottrell T. E 2001 Improved trap capture of *Euschistus servus* and *Euschistus tristigmus* (Hemiptera: Pentatomidae) in pecan orchards. Fla. Entomol. 84: 731–732.

[CIT0011] Cottrell T. E., and Horton D. 2011 Trap capture of brown and dusky stink bugs (Hemiptera: Pentatomidae) as affected by pheromone dosage in dispensers and dispenser source. J. Entomol. Sci. 46: 135–147.

[CIT0012] Cottrell T. E., Yonce C. E., and Wood B. W. 2000 Seasonal occurrence and vertical distribution of *Euschistus servus* (Say) and *Euschistus tristigmus* (Say) (Hemiptera: Pentatomidae) in pecan orchards. J. Entomol. Sci. 35: 421–431.

[CIT0013] James D. G., Heffer R., and Amaike M. 1996 Field attraction of *Biprorulus bibax* Breddin (Hemiptera: Pentatomidae) to synthetic aggregation pheromone and (E)-2-hexenal, a pentatomid defense chemical. J. Chem. Ecol. 22: 1697–1708. 24226481 10.1007/BF02272408

[CIT0014] Johnson D. T., Lewis B. A., and Mizell R. F. III 2002 Trapping brown stink bugs in peach. Horticultural studies 2001. Arkans. Agric. Exp. Stn. Res. Ser. 494: 19–23.

[CIT0015] Krupke C. H., Brunner J. F., Doerr M. D., and Kahn A. D. 2001 Field attraction of the stink bug *Euschistus conspersus* (Hemiptera: Pentatomidae) to synthetic pheromone-baited host plants. J. Econ. Entomol. 94: 1500–1505. 11777055 10.1603/0022-0493-94.6.1500

[CIT0016] Leskey T. C., and Hogmire H. W. 2005 Monitoring stink bugs (Hemiptera: Pentatomidae) in mid-Atlantic apple and peach orchards. J. Econ. Entomol. 98: 143–153. 15765676 10.1603/0022-0493-98.1.143

[CIT0017] Leskey T. C., and Hogmire H. W. 2007 Response of the brown stink bug (Hemiptera: Pentatomidae) to the aggregation pheromone, methyl (2E,4Z)-decadienoate. J. Entomol. Sci. 42: 548–557.

[CIT0018] Littell R. C., Milliken G. A., Stroup W. W., Wolfinger R. D., and Schabenberger O. 2006 SAS for mixed models, 2nd ed. SAS Institute, Cary, NC.

[CIT0019] McBrien H. L., Millar J. G., Gottlieb L., Chen X., and Rice R. E. 2001 Male-produced sex attractant pheromone of the green stink bug, *Acrosternum hilare* (Say). J. Chem. Ecol. 27: 1821–1839. 11545373 10.1023/a:1010460709535

[CIT0020] McPherson J. E., and McPherson R. M. 2000 Stink bugs of economic importance in America North of Mexico. CRS Press LLC, Boca Raton, FL.

[CIT0021] Medrano E. G., Esquivel J. F., Nichols R. L., and Bell A. A. 2009 Temporal analysis of cotton boll symptoms resulting from southern green stink bug feeding and transmission of a bacterial pathogen. J. Econ. Entomol. 102: 36–42. 19253615 10.1603/029.102.0106

[CIT0022] Mizell R. F., and Tedders W. L. 1995 A new monitoring method for detection of the stinkbug complex in pecan orchards. Proc. Southeastern Pecan Growers Association. 88: 36–40.

[CIT0023] Morrison W. R., III D-H. Lee B. D. Short A. Khrimian, and Leskey T. C. 2016 Establishing the behavioral basis for an attract-and-kill strategy to manage the invasive *Halyomorpha halys* in apple orchards. J. Pest. Sci. 89: 81–96.

[CIT0024] Sargent C., Martinson H. M., and Raupp M. J. 2014 Traps and trap placement may affect location of brown marmorated stink bug (Hemiptera: Pentatomidae) and increase injury to tomato fruits in home gardens. Environ. Entomol. 43: 432–438. 24517877 10.1603/EN13237

[CIT0025] SAS Institute 2010 SAS 9.3 for windows. SAS Institute, Cary, NC.

[CIT0026] Sugie H., Yoshida M., Kawasaki K., Noguchi H., Moriya S., Takagi K., Fukuda H., Fujiie A., Yamanaka M., Ohira Y., Tsutsumi T., Tsuda K., Fukumoto K., Yamashita M., and Suzuki H. 1996 Identification of the aggregation pheromone of the brown-winged green bug, *Plautia stali* Scott (Heteroptera: Pentatomidae). Appl. Entomol. Zool. 31: 427–431.

[CIT0027] Tedders W. L., and Wood B. W. 1994 A new technique for monitoring pecan weevil emergence (Coleoptera: Curculionidae). J. Entomol. Sci. 29: 18–30.

[CIT0028] Tillman P. G 2011 Influence of corn on stink bugs (Heteroptera: Pentatomidae) in subsequent crops. Environ. Entomol. 40: 1159–1176. 22251727 10.1603/EN10243

[CIT0029] Tillman P. G., and Cottrell T. E. 2016a Stink bugs (Hemiptera: Pentatomidae) in pheromone-baited traps near field crops in Georgia. Fla. Entomol. 99: 363–370.

[CIT0030] Tillman P. G., and Cottrell T. E. 2016b Density and egg parasitism of stink bugs (Hemiptera: Pentatomidae) in elderberry and dispersal into crops. J. Insect Sci. 16: 1–14. 27773875 10.1093/jisesa/iew091 PMC5088694

[CIT0031] Tillman P. G., and Cottrell T. E. 2017 Use of pheromones for monitoring phytophagous stink bug (Hemiptera: Pentatomidae) populations, pp. 210–225. *In* Cokl A. and Borges M. (eds.), Stinkbugs: biorational control based on communication processes, CRC Press, Boca Raton, FL.

[CIT0032] Tillman P. G., Aldrich J. R., Khrimian A., and Cottrell T. E. 2010 Pheromone attraction and cross-attraction of *Nezara*, *Acrosternum*, and *Euschistus* spp. stink bugs (Heteroptera: Pentatomidae) in the field. Environ. Entomol. 39: 610–617. 20388294 10.1603/EN09114

[CIT0033] Tillman P. G., Cottrell T. E., Mizell R. F., and Kramer E. 2014 Effect of field edges on dispersal and distribution of colonizing stink bugs across farmscapes of the southeast USA. Bull. Entomol. Res. 104: 56–64. 24044749 10.1017/S0007485313000497

[CIT0034] Tillman P. G., Khrimian A., Cottrell T. E., Lou X., Mizell R. F. III, and Johnson C. J. 2015 Trap cropping systems and a physical barrier for suppression of stink bugs (Hemiptera: Pentatomidae) in cotton. J. Econ. Entomol. 108: 2324–2334. 26453721 10.1093/jee/tov217

[CIT0035] Williams M. R 2017 Cotton insect losses – 2016 http://www.entomology.msstate.edu/resources/croplosses/2016loss.asp.

[CIT0036] Yonce C., and Mizell R. III 1997 Stink bug trapping with a pheromone. Proc. Southeastern Pecan Growers Assoc. 90: 54–56.

